# The Parametric Study of Electroosmotically Driven Flow of Power-Law Fluid in a Cylindrical Microcapillary at High Zeta Potential

**DOI:** 10.3390/mi8120344

**Published:** 2017-11-28

**Authors:** Shuyan Deng

**Affiliations:** Department of Mathematics, City University of Hong Kong, 83 Tat Chee Avenue, Kowloon, Hong Kong; sydeng4-c@my.cityu.edu.hk; Tel.: +86-147-1440-6414

**Keywords:** electroosmotic flow, power-law fluids, high zeta potential, flow behavior index, volumetric flow rate

## Abstract

Due to the increasingly wide application of electroosmotic flow in micromachines, this paper investigates the electroosmotic flow of the power-law fluid under high zeta potential in a cylindrical microcapillary for different dimensionless parameters. The electric potential distribution inside a cylindrical microcapillary is presented by the complete Poisson-Boltzmann equation applicable to an arbitrary zeta potential. By solving the Cauchy momentum equation of power-law fluids, the velocity profile, the volumetric flow rate, the average velocity, the shear stress distribution and dynamic viscosity of electroosmotic flow of power-law fluids in a cylindrical microcapillary are studied for different low/high zeta potential, flow behavior index, dimensionless electrokinetic width. The velocity profile gradually changes from parabolic to plug-like shape as the flow behavior index decreases or as the dimensionless electrokinetic width increases. For shear thinning fluids, the viscosity is greater in the center of the microchannel than that near the channel wall, the reverse is true for the shear thickening fluids. Greater volumetric rate and average velocity can be achieved by enhancing the dimensionless electrokinetic width, flow behavior index and zeta potential. It is noted that zeta potential and flow behavior index are important parameters to adjust electroosmotic flow behavior in a cylindrical microcapillary.

## 1. Introduction

Microfluidic technologies have been studied in recent years and resulted in wide applications in microelectric mechanic systems and bio-sensor areas, such as lab-on-a-chip [1,2]. The operation of microfluidic devices is completed by using the electrophoresis, electro-osmosis and other electrokinetic phenomenon [3,4,5]. The contact between the cylindrical microcapillary wall and the electrolyte leads to the exchange of charges in the tube wall and results in the formation of electric double layer (EDL) [6]. Positively charged ions gather near the wall and form the Stern layer, whose thickness is only one diameter of ions. Immediately close to the Stern layer, there is the diffuse layer which contains both the cations and anions, and the distribution of the ion density obeys the Boltzmann distribution [7]. Because of the applied electric field along the charged surface tangent direction, the free ions of the EDL migrate due to the effect of Coulomb force. Owing to the viscosity of fluid, the migration of free charged ions results in the so-called electroosmotic flow (EOF). Now the EOF is widely investigated in biological, chemical and pharmaceutical fields. Microfluidic technologies focus on building microfluidic channel system to achieve a variety of complex microfluidic manipulation functions. Micromachines are also required to include pumps, valves, mixers, filters, separators, etc. The fluid flow behavior in a microchannel is essentially different from the fluid flow behavior in everyday life with people seen, and hence micropumps, microvalves, micromixers, microfilters, separators and other micro-miniature devices compared with the corresponding macro devices vary widely. In order to accurately design microfluidic devices required in micromachines, the characteristics of fluid flow in a microchannel are discussed firstly in [8]. 

In a variety of geometric shapes of microchannel, such as parallel plate channels [9], cylindrical-shaped channel [10,11,12], circular channel [13,14], ellipse-shaped channel [15], rectangular channel [16,17,18], T-shaped channel [19] and semi-circular channel [20], fully developed Newtonian fluid in the theoretical and experimental aspects of fully developed Newtonian fluids EOF have been greatly studied.

The works reported above concern the electrokinetic effects of Newtonian fluids. In real world applications, non-Newtonian fluids are widely operated in microfluidic systems, such as blood or other polymer solution in drug delivery system. These non-Newtonian fluids show some fluid properties different from that of Newtonian fluids, for instance, the variable viscosity, normal stress effects, memory effects and hysteresis fluid properties. To characterize the non-Newtonian fluid flow through microchannel in microfluidic devices, the constitutive relation given as power-law model is used to express the connection between the shear stress and kinetic viscosity. The study of the EOF of non-Newtonian fluids is yet to be investigated adequately, because the constitutive relation of non-Newtonian fluid itself complicates the both theoretical and numerical treatment of EOF. The application of aforementioned devices requires the ability to transport, manipulate and process liquid through microchannel. Das and Chakrabotry [21,22] first studied the steady EOF of the inelastic power-law fluid. The flow behavior of blood under the sole influence of electrokinetic forces was analyzed taking into account the flow parameters as functions of blood hematocrit. The same fluid model is used to model the steady EOF in slit microchannel by Zhao et al. [23]. The analytical expression of the velocity profile, which depends on the flow behavior index has been presented. Tang et al. [24] applied Lattice Boltzmann method to simulate the EOF of the power-law fluid in a microchannel. Electroviscous effects in steady fully developed flow of a power-law liquid through a cylindrical microchannel are investigated by Bharti et al. [25]. The EOF of a viscoelastic fluid in a channel, involving the operation of physiological fluid mechanics is studied by Misra et al. [26]. The EOF of power-law fluid in a rectangular microchannel is studied in details in my previous works [27,28]. The study of EOF of power-law fluids in cylindrical microcapillarys in this work serves as the first step towards theoretical understanding the role of electroosmosis in blood flow. It is also useful to the next generation requirement in nano-scale bio-sensor technologies, as well as other micromachines.

Most works investigated EOF by solving the linear Poisson-Boltzmann equation under the situation of low zeta potential (|*ξ*| ≤ 25 mV), because assuming the low zeta potential allows the simplification of mathematical model. However, zeta potential is an important parameter to obtain various characteristics of fluid which appear at the wide range in the area of microfluidic technologies. And thus, it is significant to have zeta potential |*ξ*| > 250 mV at a microchannel wall. In this case, the linear Poisson-Boltzmann equation fails to provide the expression of electric potential and it confines many works concerning EOF. There are some studies related to high zeta potential. Vasu and De [29] studied the EOF of power-law fluid with high zeta potential in a rectangular microchannel. Zhao and Yang [30] carried out Smoluchowski velocity of the EOF of power-law fluid in a microchannel with high zeta potential. 

To author’s knowledge, no one seems to have thoroughly discussed the EOF of power-law fluid in a cylindrical microcapillary at arbitrary zeta potential due to the complexity of expression of electric potential and constitutive relation of power-low fluid. In this paper the same fluid model with the previous studies [27,28] is taken. The corresponding mathematical model was developed to describe the EOF in a cylindrical microcapillary. The complete Poisson-Boltzmann equation for EDL potential distribution compared with the previous study is solved and substituted into the modified Cauchy momentum equation of power-law fluids for EOF field. The characteristics of power-low fluid for various flow behavior index, the width ratio and arbitrary zeta potential are described in detail to guide the practical operation of microfluidic systems. Furthermore, the effects of different zeta potential on the shear stress distribution, dynamic viscosity and volumetric flow rate are analyzed with different flow behavior index.

## 2. Model Description

**Figure 1:**
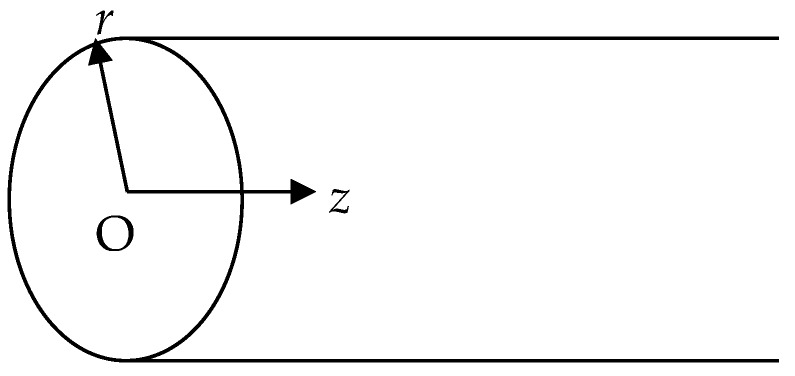
Schematic of the cylindrical microcapillary and the coordinate system used for modeling.

The EOF of power-law fluid through a cylindrical microcapillary with the *z*-axis being in the axial velocity direction is sketched in Figure 1. The coordinate system is placed in the middle of the channel. The assumptions used to formulate the mathematical model are described in detail as follows:

- According to microfluidic transport of liquid through a cylindrical microcapillary, the radius of microcapillary is much less than its length [1], and it is assumed that the flow velocity is only along the *z*-axis;
- the cylindrical microcapillary is filled with ideal symmetric electrolyte solution with constant permittivity and there is no interaction between ions [29];
- the thickness of EDL is much less than the radius of the microcapillary so that the EDLs will not overlap and the Boltzmann distribution can be applied [29];
- the wall of a microcapillary is charged with the zeta potential which is assumed to be constant [2];
- the gravitational force is negligible [2], no external pressure gradient is imposed and an electric field is applied along the central line of the microcapillary to generate EOF and
- an incompressible, laminar and fully developed flow is considered here.

### 2.1. Electrical Potential Distribution

According to the electrostatic theory, the connection between the electric potential *ψ*(*r*) of the EDL and the local net charge density $\rho_{e}$ can be expressed as Poisson equation:

(1) $$\frac{1}{r}\frac{d}{dr}(r\frac{d\psi }{dr})=-\frac{\rho_{e}}{\varepsilon \varepsilon_{0}}$$

where *ε* is the relative permittivity, $\varepsilon_{0}$ is the permittivity in vacuum and *ρ_e_* is the local net charge density. Based on the Assumptions (2) and (3), the ionic concentration per volume in electrolyte solution obeys the Boltzmann distribution:

(2) $$n_{i}=n_{i\infty }\exp\left(-\frac{z_{i}e\psi }{k_{b}T}\right)$$

where $n_{i\infty }$ is the ionic number concentration at the neutral state where *ψ*(*r*) = 0 and $n_{i}$ is the ionic number concentration of the *i*th ionic species at the state where the electric potential is *ψ*(*r*) and $z_{i}$ is the valence of the *i-*th ionic species, *e* is the elementary charge, $k_{b}$ is the Boltzmann constant and *T* is the absolute temperature. The local net charge density can be expressed as:

(3) $$\rho_{e}=e\sum n_{i\infty }z_{i}\exp\left(-\frac{z_{i}e\psi }{k_{b}T}\right)=-2en_{\infty }\sinh\left(\frac{e\psi }{k_{b}T}\right)$$

where the electrolyte is assumed to be $z_{+}$:$z_{-}$ = 1:1 and $n_{+\infty }$ = $n_{-\infty }$ = $n_{\infty }$. Substituting Equation (3) into Equation (1), one can obtain nonlinear Poisson-Boltzmann equation [10]:

(4) $$\frac{1}{r}\frac{d}{dr}(r\frac{d\psi }{dr})=-2en_{\infty }\sinh\left(\frac{e\psi }{k_{b}T}\right)$$

The boundary conditions are:

(5) $$\begin{array}{l} r=0:\frac{d\psi }{dr}=0\\ r=R:\psi =\xi \end{array}$$

where is ξ the zeta potential as given in Assumption (4). The EDL is composed of an inner layer and an outer layer. The inner layer of the EDL is known as the Stern layer where the ions are attached to the channel wall and immobile. And thus, its thickness is of one diameter of one hydrated ion. Outside the Stern layer, there is the outer layer, namely, the so-called diffuse layer where both cations and anions exist and are mobile. The shear plane between the Stern layer and the diffuse layer has an electric potential which is defined as the zeta potential.

Introducing following the dimensionless parameters, one can have:

(6) $$\begin{array}{l} \bar{r}=\frac{r}{R}\\ \bar{\psi }=\frac{e\psi }{k_{b}T}\\ K=kR\\ \bar{\xi }=\frac{e\xi }{k_{b}T} \end{array}$$

where *k* = (2*e*^2^$n_{\infty }$/$\varepsilon \varepsilon_{0}$$k_{b}$*T*)^1/2^, 1/*k* is the double layer thickness.

Substituting these dimensionless variables into Equation (4) and the boundary conditions (5), one can obtain the dimensionless Poisson-Boltzmann equation and the corresponding boundary conditions:

(7) $$\frac{1}{\bar{r}}\frac{d}{d\bar{r}}(\bar{r}\frac{d\bar{\psi }}{d\bar{r}})=K^{2}\sinh\bar{\psi }(\bar{r})$$

(8) $$\begin{array}{l} \bar{r}=0:\frac{d\bar{\psi }}{d\bar{r}}=0\\ \bar{r}=1:\bar{\psi }=\bar{\xi } \end{array}$$

An analytical solution to Equations (7) and (8), which is applicable to both low electric potential and high one, has been presented by [10]:

(9) $$\sinh\bar{\psi }\approx \{\begin{array}{cc} \bar{\psi } & \left|\bar{\psi }\right|\leq 1\\ \left|\frac{1}{2}\right|\exp\left|\bar{\psi }\right| & \left|\bar{\psi }\right|>1 \end{array}$$

(10) $${\bar{\psi }}_{L}=\frac{I_{0}(K\bar{r})}{I_{0}(K{\bar{r}}^{*})},\;0\leq \bar{r}\leq {\bar{r}}^{*}$$

(11) $${\bar{\psi }}_{H}=\ln\left\{\frac{-C}{(K\bar{r})\cos^{2}\left[\cos^{-1}\sqrt{\frac{-C}{\exp(\bar{\xi })(K^{2})}}+\frac{1}{2}\sqrt{-C}\ln(\frac{\bar{r}}{R})\right]}\right\},\;{\bar{r}}^{*}\leq \bar{r}\leq 1$$

where $I_{0}$(*r*) is the zero-order modified Bessel function, ${\bar{\psi }}_{L}$ and ${\bar{\psi }}_{H}$ are the electrical potential solutions for the low potential regime *L* and high potential regime *H* respectively, ${\bar{r}}^{*}$ is the junctions between the two regime where $\bar{\psi }$ = 1, and *C* is the integration constant determined by:

$$C={\left[2+K{\bar{r}}^{*}\frac{I_{1}(K{\bar{r}}^{*})}{I_{0}(K{\bar{r}}^{*})}\right]}^{2}-\exp(1){\left(K{\bar{r}}^{*}\right)}^{2}$$

where $I_{1}$(*r*) is the first-order modified Bessel function.

### 2.2. Equation of Motion

For power-law fluids, the shear stress distribution is a non-linear function of velocity gradient. The vector formation EOF of the Cauchy momentum equation can be derived as:

(12) $$\rho_{m}\left(\frac{\partial \vec{V}}{\partial t}+\vec{V}\cdot \nabla \vec{V}\right)=-\nabla p+\nabla \cdot \vec{\tau }+E\rho_{e}$$

where $\rho_{m}$ is the mass density, $\vec{V}$ is EOF velocity, *t* is the time, *p* is the pressure, *E* is the applied electric field strength and $\vec{\tau }$ is the shear stress distribution, which is a non-linear function of velocity gradient. 

Using Assumption (1), the flow in the microcapillary is only along the *z* direction and the velocity field is given as *u*(*r*). For a single direction EOF in a microcapillary, if the constitutive equation of power-law fluid is denoted as follow:

(13) $$\tau =\eta \frac{du}{dr}=\mu_{0}{\left(-\frac{du}{dr}\right)}^{n-1}\frac{du}{dr}=-\mu_{0}{\left(-\frac{du}{dr}\right)}^{n}$$

*η* is the viscosity of power law fluid and $\mu_{0}$ is the flow consistency index. *u*(*r*) is the velocity field along *z* axis and *n* is the flow behavior index.

Combining Assumptions (5) and (6) with continuity equation of fluid flow and substituting Equation (13) into Equation (12), the modified momentum equation can be expressed as:

(14) $$\frac{1}{r}\frac{d}{dr}(r\mu_{0}{\left(-\frac{du}{dr}\right)}^{n})=\rho_{e}E$$

From the equation above, *n* = 1 represents Newtonian fluid with constant coefficient of viscosity $\mu_{0}$. While *n* < 1 and *n* > 1 correspond to the case of shear thinning (pseudoplastic) fluids and shear thickening (dilatant) fluids, which are with variable viscosity. And the boundary conditions that Equation (14) is subject to are:

(15) $$\begin{array}{l} r=0:\frac{du}{dr}=0\\ r=R:u=0 \end{array}$$

Introducing the following dimensionless parameters:

(16) u¯=uU0Re=ρU0RμE¯=RReEξG=2n0eξρU02

where *μ* = $\mu_{0}$(*U*_0_*/R*)*^n^*^−1^, one can non-dimensionalize Equations (12) and (13) respectively:

(17) $$\frac{1}{\bar{r}}\frac{d}{d\bar{r}}[\bar{r}{\left(\frac{d\bar{u}}{d\bar{r}}\right)}^{n}]={\left(-1\right)}^{n-1}G\bar{E}\sinh\bar{\psi }(\bar{r})$$

(18) $$\begin{array}{l} \bar{r}=0:\frac{d\bar{u}}{d\bar{r}}=0\\ \bar{r}=1:\bar{u}=0 \end{array}$$

Here *U*_0_ is the reference velocity defined as ($\varepsilon \varepsilon_{0}$/$\mu_{0}$)*·E·*($k_{b}$*T*/*e*), Re is the Reynolds number, *G* represents a measure of the ability that converts the electrical energy $en_{0}\xi$, to the fluid kinetic energy, *ρU*_0_^2^/2.

Considering Equation (9), while the dimensionless zeta potential $\left|\bar{\xi }\right|\leq 1$ (namely, |*ξ*| ≤ 25 mV), the solution of Poisson-Boltzmann equation is written as:

(19) $$\bar{\psi }(\bar{r})=\frac{I_{0}(K\bar{r})}{I_{0}(K)},\;0\leq \bar{r}\leq 1$$

For Equation (17), the case of the flow behavior index *n =* 1 corresponds to Newtonian fluid flow where *η* = $\mu_{0}$, and thus Equation (17) being subject to Equation (18) can be written as:

(20) $$\bar{u}(\bar{r})=G\bar{E}\frac{\bar{\xi }}{K^{2}}\left[\frac{I_{0}(K\bar{r})}{I_{0}(K)}-1\right]$$

In the case of *n ≠* 1, Equation (17) being subject to boundary conditions Equation (18) becomes:

(21) $$\bar{u}(\bar{r})={\left[{\left(-1\right)}^{n-1}G\bar{E}\right]}^{1/n}{\int_{1}^{\bar{r}}\left[\frac{1}{t}\int_{0}^{t}x\sinh\bar{\psi }(x)dx\right]}^{1/n}dt$$

which is figured out by the numerical integral method.

From Equations (13) and (17), the dimensionless shear stress distribution is indicated as:

(22) $$\bar{\tau }(\bar{r})=\mu G\bar{E}\cdot \frac{1}{\bar{r}\tau_{w}}\int_{0}^{\bar{r}}x\sinh\bar{\psi }(x)dx$$

(23) $$\tau_{w}=\mu G\bar{E}\cdot \int_{0}^{1}\bar{r}\sinh\bar{\psi }(\bar{r})d\bar{r}$$

And the expression for the dimensionless dynamic viscosity of the liquids is given as the following form:

(24) $$\bar{\eta }(\bar{r})=\frac{\mu }{\eta_{w}}{\left[{\left(-G\bar{E}\right)}^{1/n}\cdot {\left(\frac{1}{\bar{r}}\int_{0}^{\bar{r}}x\sinh\bar{\psi }(x)dx\right)}^{1/n}\right]}^{n-1}$$

(25) $$\eta_{w}=\mu {\left[{\left(-G\bar{E}\right)}^{1/n}\cdot {\left(\int_{0}^{1}\bar{r}\sinh\bar{\psi }(\bar{r})d\bar{r}\right)}^{1/n}\right]}^{n-1}$$

The volumetric flow rate and dimensionless average velocity through the cross section of the microcapillary are respectively given as:

(26) $$Q=2\pi \int_{0}^{1}\bar{r}\cdot \bar{u}(\bar{r})d\bar{r}$$

(27) $${\bar{u}}_{avr}=\frac{Q}{\pi }$$

From Equations (26) and (27), the magnitude of average velocity is proportional to the volumetric flow rate. When the flow behavior index *n =* 1, the volumetric flow rate and average velocity of Newtonian fluid can be carried out theoretically.

## 3. Results

The parametric values are taken as follows. The strength of electric field is *E* = 10,000 V/m and the concentration of electrolyte solution $n_{0}$ = *N*_A_*c* where *c* is the molar concentration and *N*_A_ = 6.02 × 10^23^/mol, *e* = 1.6 × 10^−19^, $k_{b}$ = 1.38 × 10^−23^ J/K, *ρ =* 1000 kg/m^3^, *μ =* 9 × 10^−4^ m^2^/s, *ε* = 80, $\varepsilon_{0}$ = 8.85 × 10^−12^ C^2^/ J·m *T =* 293 K, *R =* 100 μm. The dimensionless zeta potential varies from −1 to −8 and the dimensionless electrokinetic width *K* ranges from 10 to 50. 

**Figure 2:**
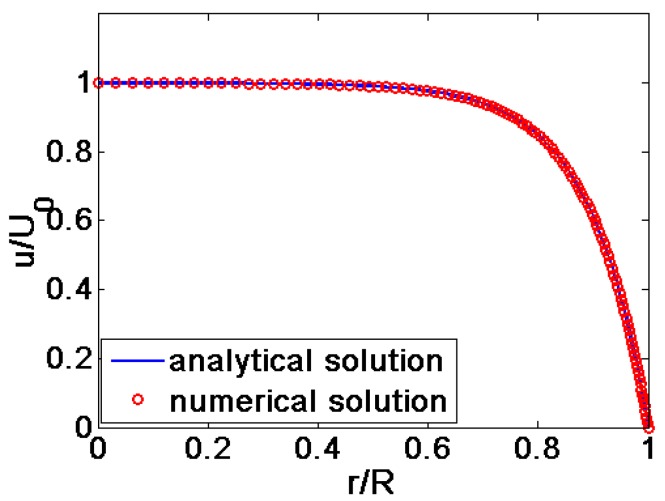
The comparison of the analytical solution with the numerical solution (*n =* 1 and $\bar{\xi }$ = −1).

**Figure 3:**
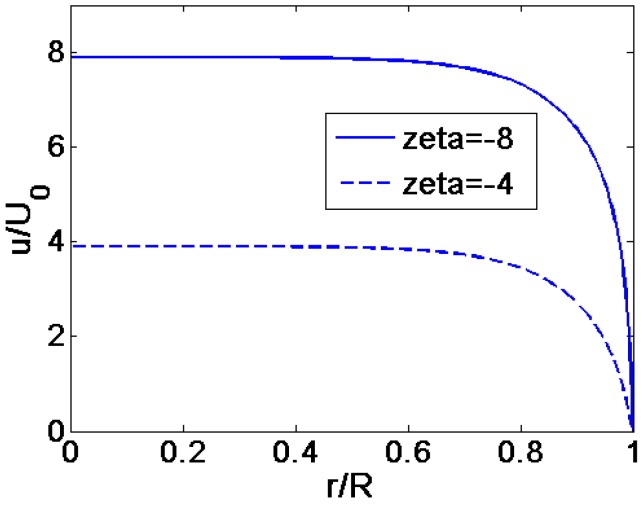
The comparison of the velocity profiles for different zeta potential (*n =* 1, *K =* 10), where the reference velocity is defined as *U*_0_ = ($\varepsilon \varepsilon_{0}$/$\mu_{0}$)*·E·*($k_{b}$*T*/*e*).

When the flow behavior index *n =* 1 and the dimensionless zeta potential $\bar{\xi }=-1$, the numerical solution of the velocity profile obtained by the numerical integral method is compared with the analytical solution given by Equation (20). It can be seen from Figure 2 that the numerical result agrees well with the theoretical result. In addition, as shown in Figure 3, the reference velocity is taken as the form of *U*_0_ = ($\varepsilon \varepsilon_{0}$/$\mu_{0}$)*·E·*($k_{b}$*T*/*e*) so that the effects of different high zeta potential $\bar{\xi }$ can be shown clearly. And it is observed that the results of Kang [10] are in good agreement with the numerical results of the present work at higher zeta potential. Therefore, the numerical method of the present work can precisely predict the characteristics of power-low fluid flow under the high zeta potential. Using the same method, the velocity profiles are computed for shear thinning (*n* < 1) and shear thickening fluids (*n >* 1). Furthermore, the dimensionless viscosity distribution of power-law fluids across the quarter cross section of the microcapillary and dimensionless shear stress profile are presented for various width ratio *K*. Based on the results above, the dimensionless average velocity and volumetric flow rate for different parameters are computed.

**Figure 4:**
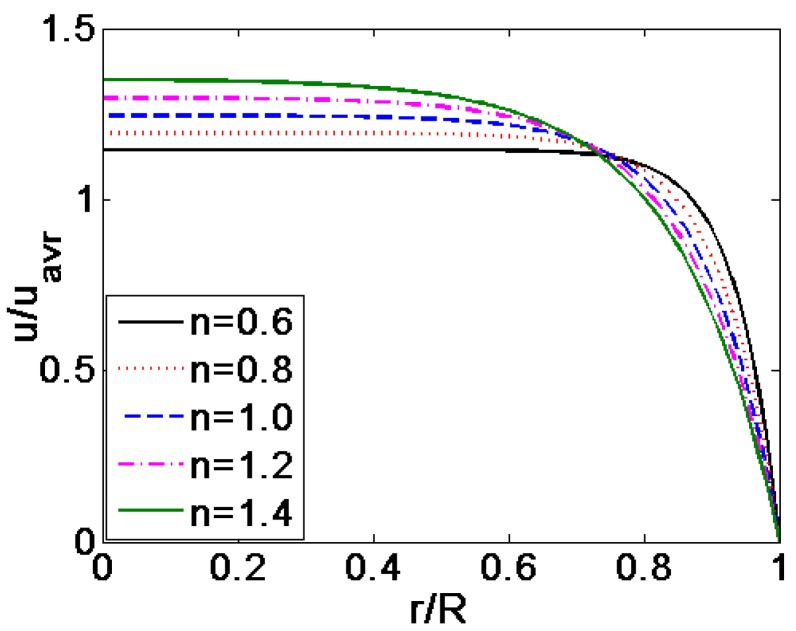
The comparison of the dimensionless velocity profiles generalized with the respective dimensionless average velocity for various flow behavior index *n* (*K =* 10, $\bar{\xi }$ = −4).

**Figure 5:**
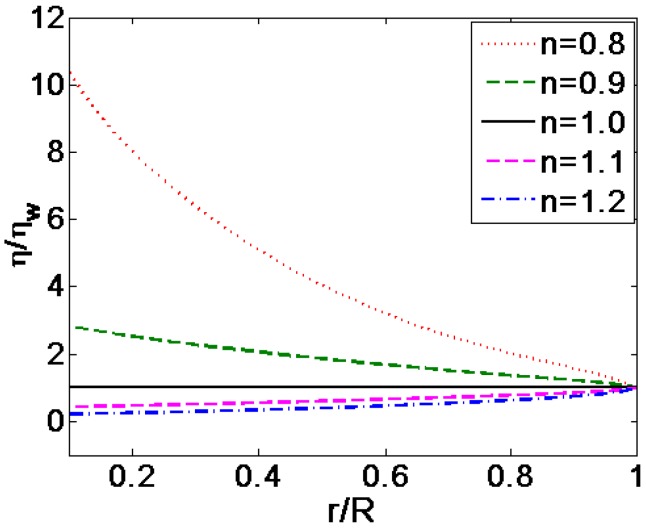
The comparison of dimensionless dynamic viscosity distributions generalized with the respective magnitude of dynamic viscosity at the channel wall $\eta_{w}$ for various values of flow behavior index *n* (*K* = 10, $\bar{\xi }$ = −4).

Figure 4 shows the comparison of the velocity profiles for various flow behavior index *n*, where the velocities are generalized with the respective average velocity. Figure 5 is the variation of dynamic viscosity distribution generalized by the viscosity of Newtonian fluid related to the change in flow behavior index *n*, where *K* = 10, $\bar{\xi }$ = −4. It can be seen from Figure 4 that the pattern of velocity profiles depend greatly on the fluid behavior index *n*. For shear thinning fluids, namely *n <* 1, the velocity profile becomes more plug-like with the decrease of the flow behavior index *n*. This is, as shown in Figure 5, due to the fact that the dynamic viscosity increases as a whole when the value of flow behavior index *n* decreases. And for the shear thickening fluids, namely *n <* 1, the viscosity reaches high magnitude close to the center of the channel, which decreases along the radial direction and decrease to unity at the channel wall finally. For the shear thickening fluids, namely *n >* 1, the velocity profile varies from parabolic to plug-like shapes as *n* approaches to 1. This is because that viscosity as a whole increases gradually as the flow behavior index *n* decreases and the magnitude of viscosity shows gradual increase along the radial direction from the center to the wall of microcapillary and finally reaches unity at the channel wall as shown in Figure 5. It is worth mentioning that the value of viscosity is infinite following the decrease of the value of *n* in the absence of dimensionless velocity gradient.

**Figure 6:**
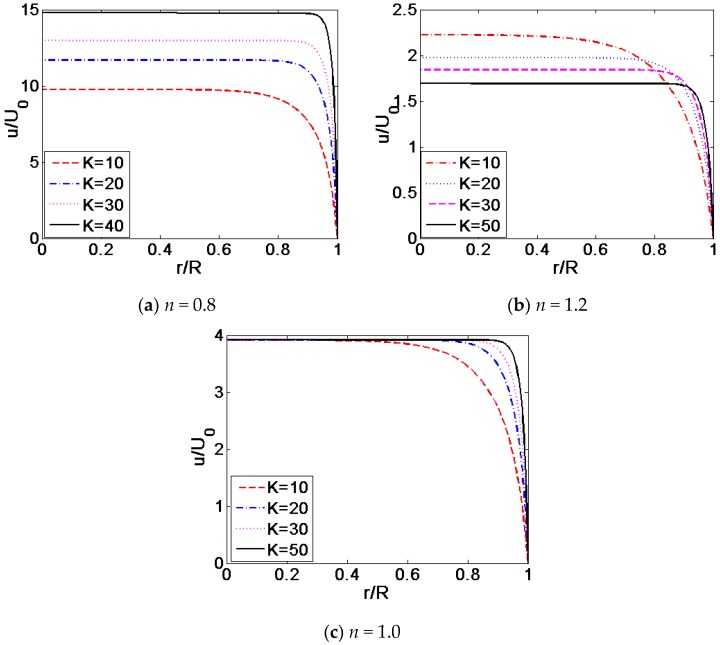
The comparison of the velocity profiles for various width ratio *K* under the situation of various flow behavior index *n* ($\bar{\xi }$ = −4): (**a**) *n* = 0.8; (**b**) *n* = 1.2; (**c**) *n* = 1.

**Figure 7:**
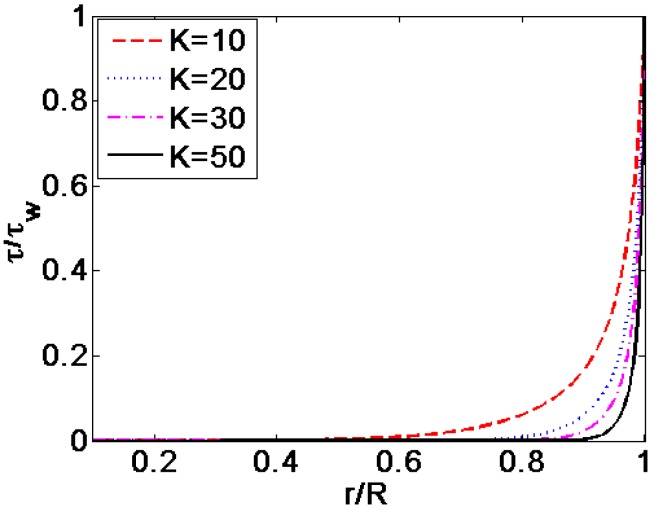
The corresponding changes of dimensionless shear stress distribution generalized with the magnitude of shear stress at the channel wall $\tau_{w}$ for various width ratio *K* ($\bar{\xi }$ = −4).

Figure 6 described the variation of the velocity profiles, with *K* for different flow behavior index *n*: (a) *n* = 0.8, (b) *n* = 1.2 and (c) *n* = 1. Figure 7 described the variation of the shear stress distribution for different dimensionless electrokinetic width *K*. It can be seen from Figure 6 that regardless of the value of flow behavior index *n*, the velocity profile becomes more plug-like as the value of *K* transits from 10 to 50, while keeping other parameters unchanged where $\bar{\xi }$ = −4. Enhancing the dimensionless electrokinetic width *K* indicates that the ratio of the radius of microcapillary *R* to the double layer thickness 1/*k* increases. Namely, the density of free charged ions decreases relatively. With the increase of *K*, the free ions only exist in the region close to the channel wall. As shown in Figure 7, the shear stress near the channel wall changes slowly when *K* = 10 and shows rapid change when width ratio *K* reaches 50. When dimensionless electrokinetic width *K* gets larger and larger, the dimensionless shear stress is almost equal to zero in the entire region of the channel except for the channel wall where the shear stress distribution rapidly jumps to a certain magnitude, leading to the changes in the velocity profile near the channel wall. Furthermore, the magnitude of dimensionless shear stress is independent on the flow behavior index *n* from Equations (22) and (23).

**Figure 8:**
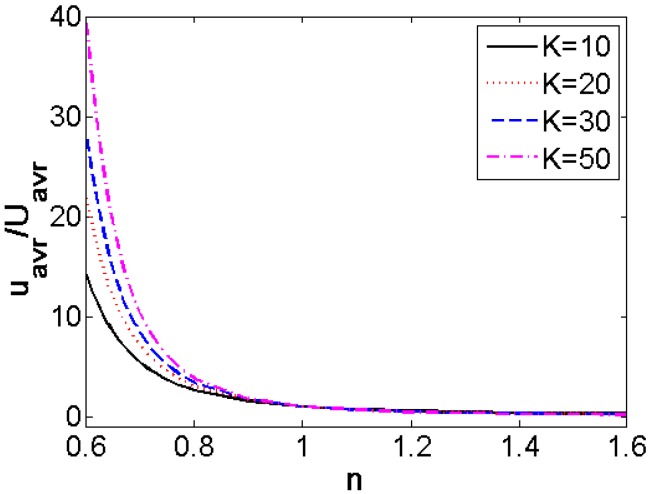
The variation of dimensionless average velocity generalized with the average velocity of Newtonian fluid (*n =* 1) with flow behavior index *n* under the situation of different width ratio *K* ($\bar{\xi }$ = −4).

Figure 8 described the variation of dimensionless average velocity generalized with the average velocity of Newtonian fluid (*n* = 1) when the flow behavior index *n* changes from 0.6 to 1.6 under the situation of different width ratio *K*. It is noted that for shear thinning fluids the dimensionless average velocity is much more times higher than that of Newtonian fluid. It is more and more apparent with the decrease of *n*. The increase in the width ratio *K* leads to the increase in the dimensionless average velocity. It gets more evident for the lower flow behavior index *n*. In other words, the dimensionless average velocity increases as the flow behavior index *n* decreases. And when *n >* 1, the variation of dimensionless average velocity is slight especially for higher flow behavior index *n*. The change in the width ratio *K* and the change in the flow behavior index *n* show little influence on the dimensionless average velocity of EOF. Therefore, the EOF of shear thinning fluid is much more sensitive to the change in *K* and *n* than that of shear thickening fluids. It is worth mentioning that high average velocity and high volumetric flow rate can be obtained by enhancing the width ratio *K* or reducing the flow behavior index *n*.

**Figure 9:**
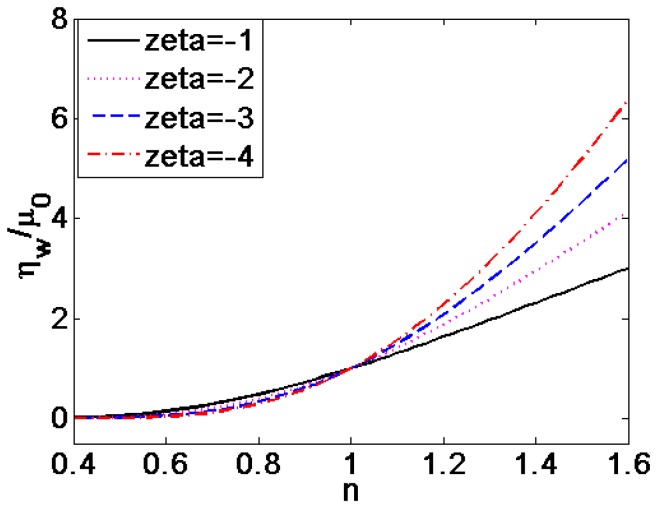
The variation of dimensionless dynamic viscosity at the channel wall generalized with the viscosity of Newtonian fluid $\mu_{0}$ with flow behavior index *n* under the situation of different dimensionless zeta potential $\bar{\xi }$ (*K* = 10).

Figure 9 described the influence of different dimensionless zeta potential on dimensionless dynamic viscosity at the channel wall, which is generalized by the dynamic viscosity of Newtonian fluid. It is found that the dimensionless dynamic viscosity increases with the increase of zeta potential. In addition, the influence of zeta potential gets greater at higher flow behavior index *n* for the shear thickening fluids. In contrast, when *n* < 1 for shear thinning fluids, the influence of zeta potential on the dynamic viscosity at the channel wall is insignificant.

**Figure 10:**
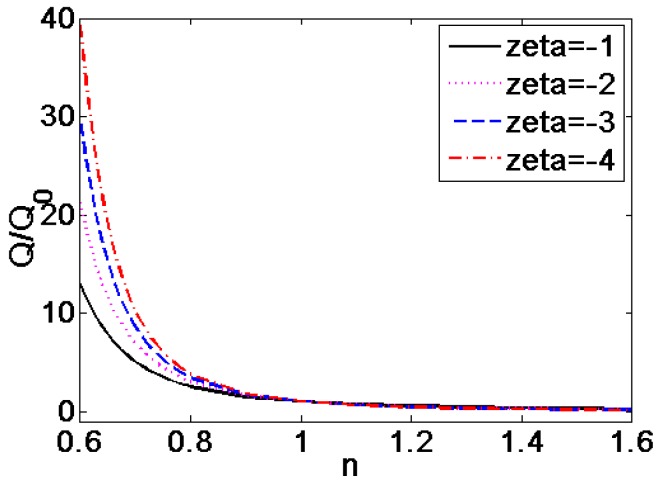
The variation of dimensionless volumetric flow rate generalized with the volumetric flow rate of Newtonian fluid (*n* = 1) with flow behavior index *n* under the situation of different zeta potential $\bar{\xi }$ (*K* = 50).

In Figure 10, the influence of different dimensionless zeta potential on the dimensionless volumetric flow rate generalized with the volumetric flow rate of Newtonian fluid (*n* = 1) is described where *K* = 10. It is observed that the dimensionless volumetric flow rate is more than ten times higher than that of the Newtonian fluid and shear thinning fluids, and this effect gets greater at the higher zeta potential and the lower flow behavior index *n*. For the shear thickening fluids, the effect of dimensionless zeta potential on the volumetric flow rate is insignificant, compared with the case for shear thinning fluids.

## 4. Conclusions

This paper presented a detailed study for the EOF of power-law fluid in a cylindrical microcapillary under high zeta potential. The electric potential distribution in the flow field is given by the complete Poisson-Boltzmann equation which is nonlinear and solved under the situation of both low and high zeta potential. Using the numerical integral method, the semi-analytical solution of Cauchy momentum equation for power-low fluids is carried out. And the numerical dimensionless velocity profile and dynamic viscosity distribution for various flow behavior index *n* and high zeta potential are obtained. The numerical dimensionless velocity profile and corresponding shear stress distribution are computed for different width ratio *K*. It is found that the flow behavior index *n* and the dimensionless electrokinetic width *K* (width ratio) play important roles for adjusting the EOF pattern. The shapes of velocity profiles change from parabolic to plug-like as the flow behavior index *n* decreases or the width ratio gets higher. For shear thinning fluids, the viscosity decreases along the radial direction from the center to the microcapillary wall and the reverse is true for shear thickening fluids. Furthermore, it is found that the high zeta potential is strongly responsible for the increase of the magnitude of average velocity. The method of attaining higher average velocity or volumetric flow rate is presented by changing the flow behavior index *n*, width ratio *K* or the zeta potential *ξ*. Therefore, in the area of practical application, adjusting zeta potential, dimensionless electrokinetic width and flow behavior index can be achievable method to operate the EOF in micromachines [23]. For example, to achieve a plug-like shape for velocity profile, the working fluid in microfluidic systems can be chosen as shear thinning fluids rather than Newtonian fluids or shear thickening fluids. When designing the microfluidic systems, it is notable that the variation in electrokinetic width resulting from choosing different electrolyte solution or different radius of the microcapillary plays an important role to the shape of velocity profile and volumetric flow rate across the cross section of the microcapillary. Besides, different zeta potential has significant influence on the average velocity and volumetric flow rate, therefore, one should have second thought about always assuming low zeta potential when characterizing the EOF behavior in micromachines. To transport fluids in microfluidic device efficiently, adjusting electrokinetic width or zeta potential is more achievable method than enhancing the electric field strength.
